# Wideband low-RCS and gain-enhanced antenna using frequency selective absorber based on patterned graphene

**DOI:** 10.1038/s41598-024-60143-1

**Published:** 2024-04-23

**Authors:** Fuwei Wang, Yi Wang, Xiaoyu Zhang, Lu Liu, Ke Li, Yuhui Ren

**Affiliations:** School of Information Technology, Northwest University Xi’an, Shaanxi, 710127 China

**Keywords:** Electrical and electronic engineering, Information technology

## Abstract

In this paper, a double-layer patterned graphene-based frequency-selective absorber (DGFSA) is proposed as a means of reducing an antenna’s radar cross-section (RCS) while simultaneously increasing its gain. The antenna consists of a patch antenna with Multi-Graphene Frequency Selective Absorber (MGFSA) mounted on top. The DGFSA consists of double-layer patterned graphene and a band-pass frequency selective surface (FSS). Two patterned graphene lossy layers with different square resistances are used, which broaden the electromagnetic (EM) wave absorption bandwidth of the DGFSA, thus greatly reducing the out-band monostatic RCSs of the patch antenna. Meanwhile, due to the quasi-Fabry-Perot (F-P) effect, the gain of the proposed antenna is enhanced by 2.4 dB. Additionally, the low-RCS antenna reduces the monostatic RCS from 1.32 to 17 GHz under y-polarization and from 1.4 to 16.8 GHz under x-polarization, respectively. Furthermore, a decrease in the bistatic RCS is accomplished. Results from simulations and measurements match up nicely, which means the antenna we proposed has a good application on the stealth platform.

## Introduction

It is common knowledge that the antenna has a high contribution to the radar cross-section (RCS) of the platform equipment^1^. And it’s not easy to reduce an antenna’s RCS without affecting its primary radiating properties.

Due to the outstanding properties of metasurfaces in EM wave amplitude and phase modification, metasurfaces have recently found widespread application in antenna RCS reduction^2^. For instance, chessboard-layout metasurfaces were utilized to reduce the RCS of an antenna by cancelling the phase of the incident and reflected waves^3–6^, what’s more, to lower the RCS of the antennas, researchers employed polarization conversion metasurfaces.^2–10^. However, this method is effective for monostatic RCS. Therefore, researchers loaded antennas with metamaterial absorbers^11–13^. In this way, monostatic RCS can be reduced, and the bistatic RCSs can also be well reduced. But the antenna will suffer from unexpected radiation loss and the RCS reduction band is narrow.

To solve the above problems of metamaterial absorbers, frequency selective absorbers (FSA) are proposed^14–18^. FSA can ensure the normal radiation of the antenna while absorbing EM waves in broadband. However, due to the insertion loss of FSAs, the majority of FSAs are incapable of improving the gain of the antenna and reducing its RCS at the same time^19^.

Regarding existing FSA designs for antenna RCS reduction, the bulk of published structures employ lumped resistors for absorption. Although lumped resistors are lightweight and simple to obtain, they are susceptible to parasitic effects and have limited power handling capacities^20^. At the same time, it is difficult to weld massive small lumped resistors.

Graphene is a unique 2-D material with excellent light transmittance, high heat conductivity, and high electron mobility^21^. The majority of terahertz absorbers, for instance, have been proposed^22,23^. Recently, due to the dispersionless and tunable resistance in the microwave band, absorbers based on graphene have been proposed.^24–27^. In^25^, A graphene-based microwave absorber with dynamic tuning is proposed, the centre frequency of the absorption band can be tuned, while the absorption performance remains unchanged. Inspired by^25^, in^27^, a hybrid structure of graphene and indium tin oxide (ITO) is presented, which can function as an optically transparent, frequency-tunable absorber. The absorber employs ITO film to replace the traditional absorber’s metal floor, thereby achieving optical transparency. However, few absorbers based on graphene are used to reduce the RCS .

In^28^, a via-based hybrid metal-graphene metamaterial absorber is proposed. To evaluate the absorption effectiveness of the proposed absorber, the author compares the RCS of the absorber to that of a metal plate when EM waves are incident from various angles. The simulation results reveal that the RCS reduces marginally from 2 to 12 GHz. According to our best knowledge, there are few designs for reducing the wide-band RCS of antennas utilizing absorbers based on patterned chemical vapour deposition (CVD) graphene.

In this paper, a low-RCS and gain-enhanced antenna that utilizes FSA based on double-layer graphene (DGFSA) is proposed. The proposed DGFSA uses two graphene lossy layers with different square resistances, with the upper layer’s square resistance of the top being 1200 and the second layer’s square resistance being 50. Thus, the proposed DGFSA has a broad absorption bandwidth for EM waves. The MGFSA that we proposed has a wide-band EM wave absorption bandwidth. By utilizing the reflecting property of the DGFSA superstrate, an F-P cavity can be produced between the DGFSA and the antenna’s ground, hence improving the antenna’s gain by 2.4 dB. With the DGFSA’s wide-band absorption, both x- and y- polarization of the antenna could obtain wide-band monostatic RCS reduction (169.2% fractional bandwidth, FBW), and bistatic RCS reduction is also realized.

This paper is arranged as follows: Section “Design of Proposed DGFSA” proposes a DGFSA with broad-band absorption. The DGFSA is discussed using the transmission line (TL) model and the performance of DGFSA is presented. In Section “Design of proposed low-RCS and gain enhancement antenna”, we present the low-RCS and gain enhancement antenna based on MGFSA and the radiation and scattering characteristics of the proposed antenna are studied. In Section “Experimental verification”, the measured results of the prototype antenna are presented. In Section “Conclusion”, a summary of the conclusion is presented.

## Design of proposed DGFSA

**Figure 1:**
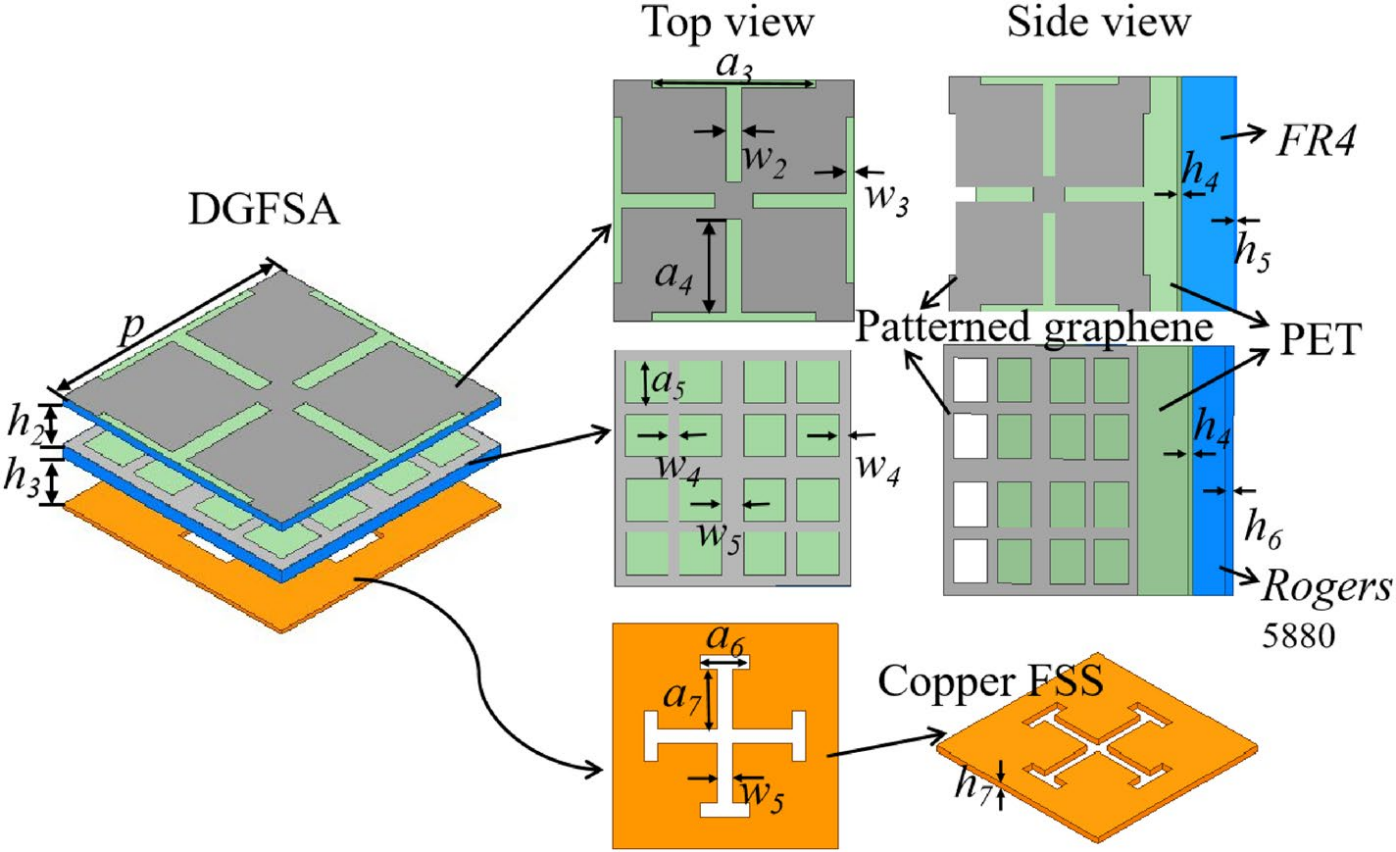
The unit cell of proposed DGFSA. (Geometrical parameters: *p* = 32 mm, $h_{2}$ = 5 mm, $h_{3}$ = 5.4 mm, $a_{3}$ = 22 mm, $w_{2}$ = 2 mm, $w_{3}$ = 1 mm, $a_{4}$ = 12.5 mm, $a_{5}$ = 5.75 mm, $w_{4}$ = 1.5 mm, $w_{5}$ = 3 mm, $a_{6}$ = 7 mm, $a_{7}$ = 8.5 mm, $w_{5}$ = 2 mm, $h_{4}$ = 0.125 mm, $h_{5}$ = 1 mm, $h_{6}$ = 1.6 mm, $h_{7}$ = 0.5 mm).

In Fig. 1 we can see the unit cell for the DGFSA developed for this paper. The unit cell consists of double graphene-based lossy layers and a 0.5 mm thick copper band-pass frequency selective surface (FSS). The substrates used are the FR4 dielectric ($\epsilon _{FR4}$= 4.4, $tan\delta _{FR4}$= 0.02) and Rogers 5880 dielectric ($\epsilon _{Rogers}$= 2.2, $tan\delta _{Rogers}$= 0.0009) for supporting the top and second graphene layer respectively. The graphene is grown on the 50 $\mu m$-thick copper using CVD and transferred onto the polyethene terephthalate(PET) ($\epsilon _{PET}$= 3.8, $\epsilon _{PET}$ = 0.018) substrate through the lamination and etching process. In section “Design of proposed low-RCS and gain enhancement antenna”, this procedure will be illustrated in detail. The square resistance of graphene is 1200 $\Omega /sq$ for the top graphene layer and 50 $\Omega /sq$ for the second graphene layer.

### Analysis of DGFSA

**Figure 2:**
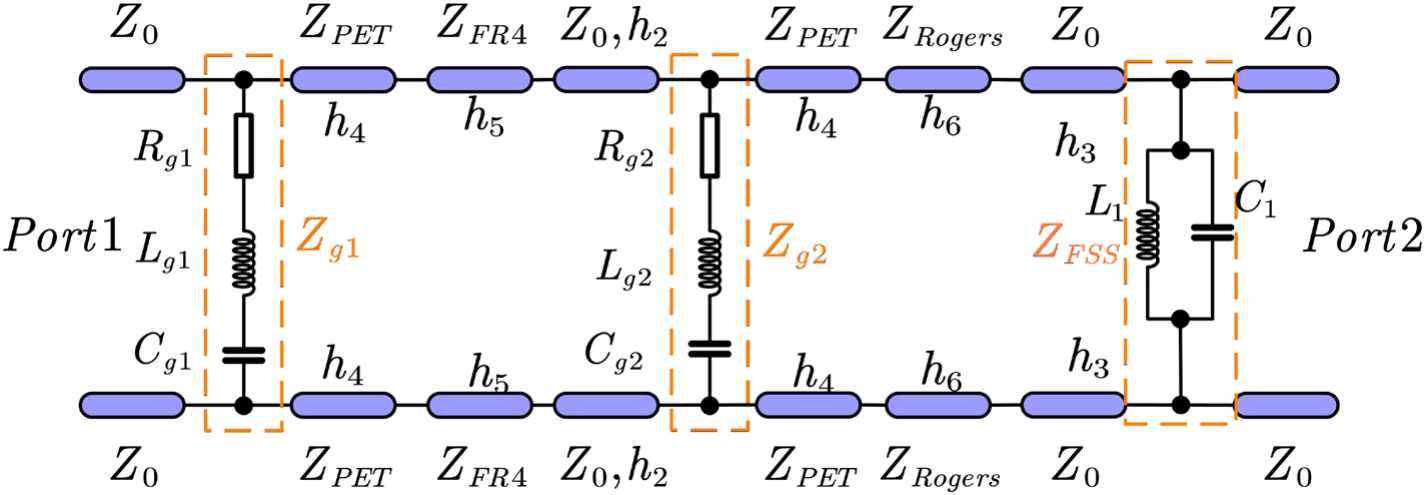
The transmission line (TL) model of the DGFSA.

We investigate MGFSA using the transmission line model to further demonstrate its working principle. Figure 2 displays the TL model to illustrate the suggested DGFSA’s working mechanism^27^.

The equivalent impedance of patterned monolayer graphene may be expressed as^25^

1 $$\begin{aligned} {Z_g} = {R_g} -j\frac{{1-{{\omega }^2L_gC_g}}}{{\omega }C_g} \end{aligned}$$

2 $$\begin{aligned} {Z_{FSS}} = \frac{j{\omega }L_1}{1-{\omega }^2L_1C_1} \end{aligned}$$

The ${L_g}$, ${C_g}$, ${R_g}$ represent the distributed inductance, distributed capacitance and equivalent resistance of patterned monolayer graphene respectively. So, the equivalent impedance of top and second monolayer patterned graphene can be written as ${Z_{g1}}$ and ${Z_{g2}}$. We can think of the PET substrates as transmission lines of length $h_{4}$ and characteristic impedance $Z_{PET}=Z_0/{\sqrt{\epsilon _{PET}}}$, where ${Z_0}$ is the impedance of free space. At the same time, the impedance of FR4 and Rogers 5880 substrate can be written as $Z_{FR4}=Z_0/{\sqrt{\epsilon _{FR4}}}$ and $Z_{Rogers}=Z_0/{\sqrt{\epsilon _{Rogers}}}$ respectively^27^. The following is a definition of the DGFSA transmission matrix^27^:

3 $$\begin{aligned} \begin{gathered} {\left[ \begin{array}{ll} A &{} B \\ C &{} D \end{array}\right] =} \\ {\left[ \begin{array}{cc} 1 &{} 0 \\ \frac{1}{Z_{91}} &{} 1 \end{array}\right] \left[ \begin{array}{cc} \cos \beta _1 h_4 &{} j Z_{P E T} \sin \beta _1 h_4 \\ j \frac{1}{Z_{P E T}} \sin \beta _1 h_4 &{} \cos \beta _1 h_4 \end{array}\right] }\\ {\left[ \begin{array}{cc} \cos \beta _2 h_5 &{} j Z_{F R 4} \sin \beta _2 h_5 \\ j \frac{1}{Z_{F R A}} \sin \beta _2 h_5 &{} \cos \beta _2 h_5 \end{array}\right] \left[ \begin{array}{cc} 1 &{} 0 \\ \frac{1}{Z_{92}} &{} 1 \end{array}\right] } \\ {\left[ \begin{array}{cc} \cos \beta _1 h_4 &{} j Z_{P E T} \sin \beta _1 h_4 \\ j \frac{1}{Z_{P E T}} \sin \beta _1 h_4 &{} \cos \beta _1 h_4 \end{array}\right] } \\ {\left[ \begin{array}{cc} \cos \beta _3 h_6 &{} j Z_{P E T} \sin \beta _3 h_6 \\ j \frac{1}{Z_{P E T}} \sin \beta _3 h_6 &{} \cos \beta _3 h_4 \end{array}\right] \left[ \begin{array}{cc} 1 &{} 0 \\ \frac{1}{Z_{P S S}} &{} 1 \end{array}\right] } \end{gathered} \end{aligned}$$

where $\beta _4$, $h_4$, $\beta _2$, $h_5$, $\beta _3$ and $h_6$ are the wave propagation constant and thickness of FR4 and Rogers 5880, respectively. Finally, the transmission coefficient and reflection coefficient of the two ports can be determined by the transmission (ABCD) matrix as follows^29^:

4 $$\begin{aligned}{} & {} T = \left| S_{21}\right| =\left| \frac{2{(A_0D_0-B_0C_0)}}{A_0+B_0+C_0+D_0}\right| \end{aligned}$$

5 $$\begin{aligned}{} & {} \quad \Gamma = \left| S_{11}\right| =\left| \frac{{A_0+B_0-C_0-D_0}}{A_0+B_0+C_0+D_0} \right| \end{aligned}$$

Where $A_0$, $B_0$, $C_0$ and $D_0$ are normalized elements of the transmission (ABCD) matrix.

**Figure 3:**
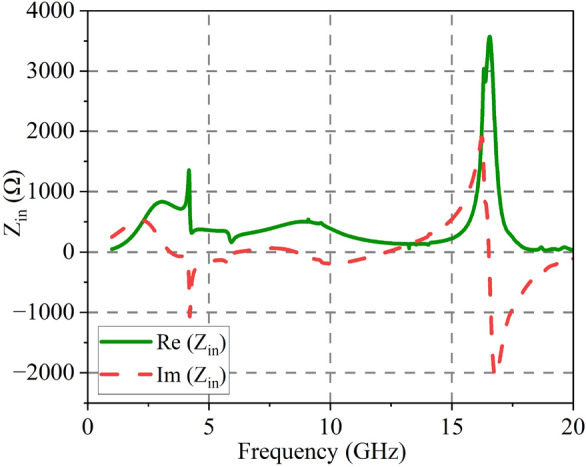
Input impedance of the proposed DGFSA.

Figure 3 presents the real part of $Z_{in}$ and the imaginary part of $Z_{in}$. The data reveals that between 3.95 and 11 GHz the real part of $Z_{in}$ is basically stable near 377 $\Omega$, and the imaginary part is stable near 0, so the reflection coefficient can be expressed as

6 $$\begin{aligned} \Gamma =\frac{Z_{in}-Z_0}{Z_{in}+Z_0} \end{aligned}$$

Thus, the proposed DGFSA is well-matched. When an electromagnetic wave incident, the graphene lossy layer will generate induced current, which will absorb the majority of electromagnetic energy.

### Performance of DGFSA

**Figure 4:**
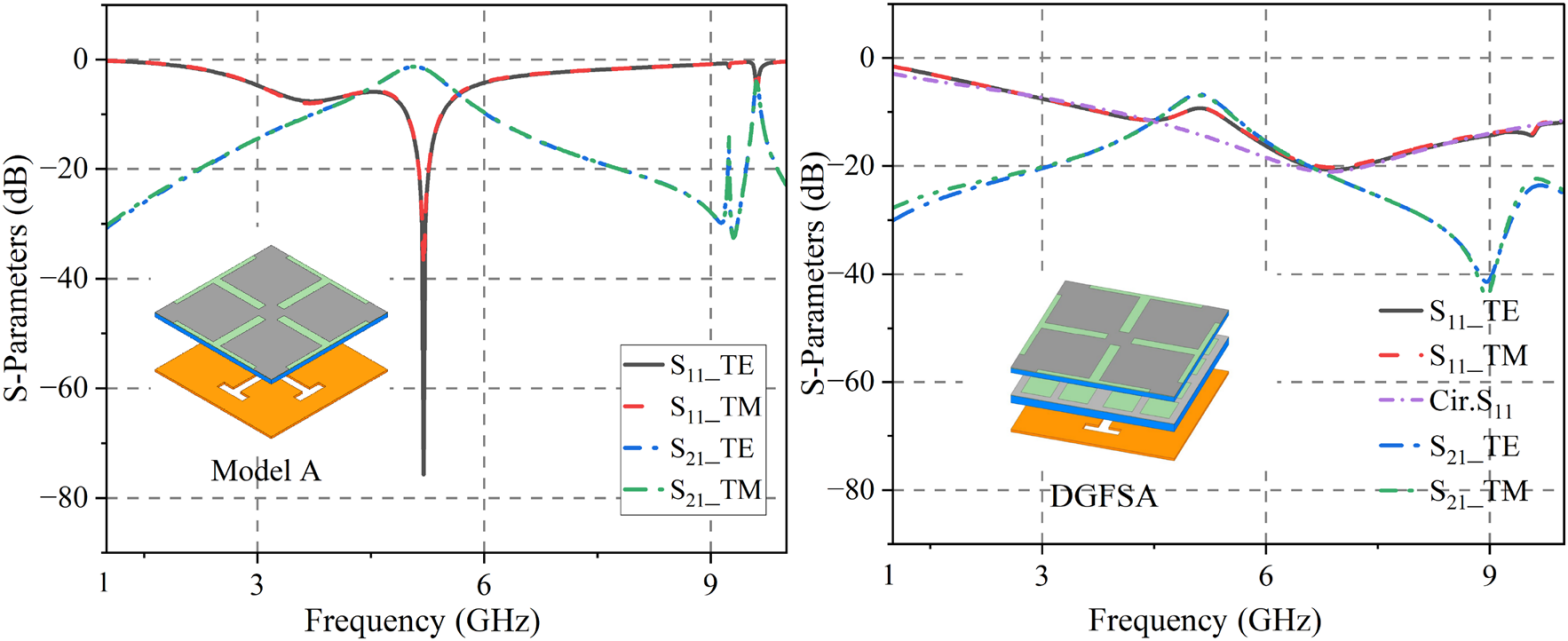
Simulated and calculated S-Parameters of Model A and DGFSA . (**a**) Model A (**b**) DGFSA.

The proposed DGFSA is presented in Fig. 1 . To elucidate the absorption mechanism of the proposed DGFSA, the |$S_{11}$| of various combinations of graphene and band-pass FSS are analyzed, and the simulation results are depicted in Fig. 4. When only the top graphene impedance layer is present (Model A), the absorption bandwidth ranges from 4.98 GHz to 5.44 GHz (|$S_{11}|<= -10$ dB), indicating a narrow absorption bandwidth. When the second graphene layer is applied, the absorption bandwidth increases from 3.9 GHz to 11.57 GHz (|$S_{11}|<= -10$ dB). Therefore, the use of various square-resistance graphene broadens the absorption bandwidth. At the same time, the |$S_{11}$| of DGFSA between equivalent circuit model (ECM) and full-wave simulations are matched well. The optimization parameters of the ECM are as follows: $R_{g1}$ = 589 $\Omega$, $L_{g1}$ = 0.72 nH, $C_{g1}$ = 0.86 pF, $R_{g2}$  = 44.5 $\Omega$, $L_{g2}$ = 3.195 nH, $C_{g2}$ = 39.31 pF, $L_1$ = 3.16 nH, $C_1$ = 0.34 pF.

To explain the principle that antenna radiation characteristics remain unchanged. The |$S_{21}$| of the DGFSA and Model A is shown in Fig. 4. The DGFSA creates a transmission band at about 5 GHz. However, the transmission performance is generally poor. The structure composed of the top graphene impedance layer and the band-pass FSS (Model A) can make a transparent window from 4.75 to 5.37 GHz (|$S_{21}|>-3$ dB), which can let the in-band electromagnetic waves of the antenna pass through. When the DGFSA is placed above the antenna, an $a_2*a_2$ slot is cut into the second graphene impedance layer to verify that the antenna radiates normally, as shown in Fig. 6.

**Figure 5:**
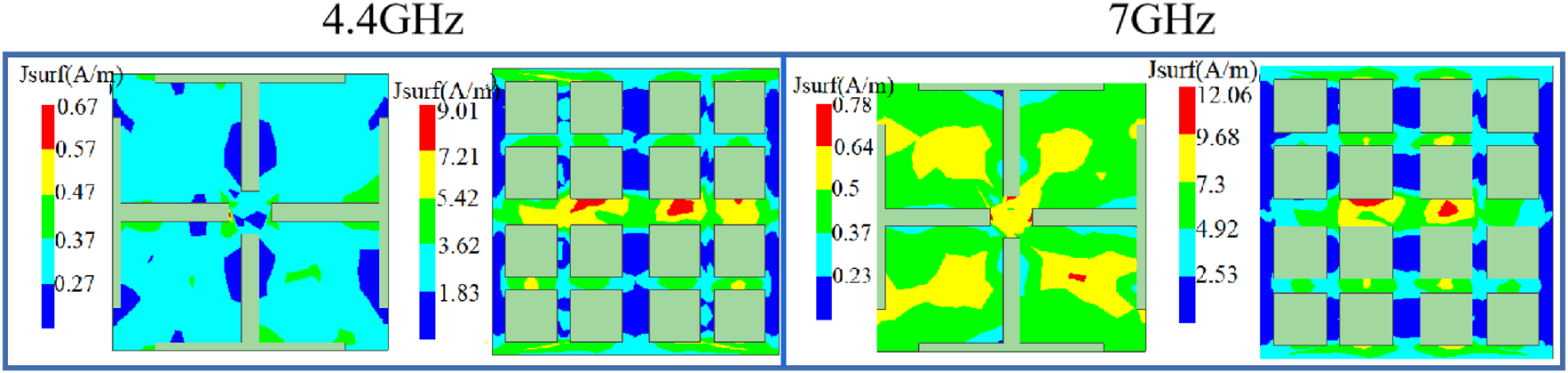
Surface current distribution of DGFSA.

The graphene impedance layer’s surface current distribution is depicted in Fig. 5 over a range of absorption frequencies. From the figure, the surface current excited by the top graphene impedance layer is relatively small, mainly absorbed by the horizontal central strip of the bottom graphene layer at 4.3 GHz. Surface current distribution at 7 GHz suggests that current in the top graphene impedance layer is highly concentrated in four squares and in the second graphene impedance layer, current is highly concentrated in other strips parallel to the horizontal central strip.

## Design of proposed low-RCS and gain enhancement antenna

### Antenna structure

**Figure 6:**
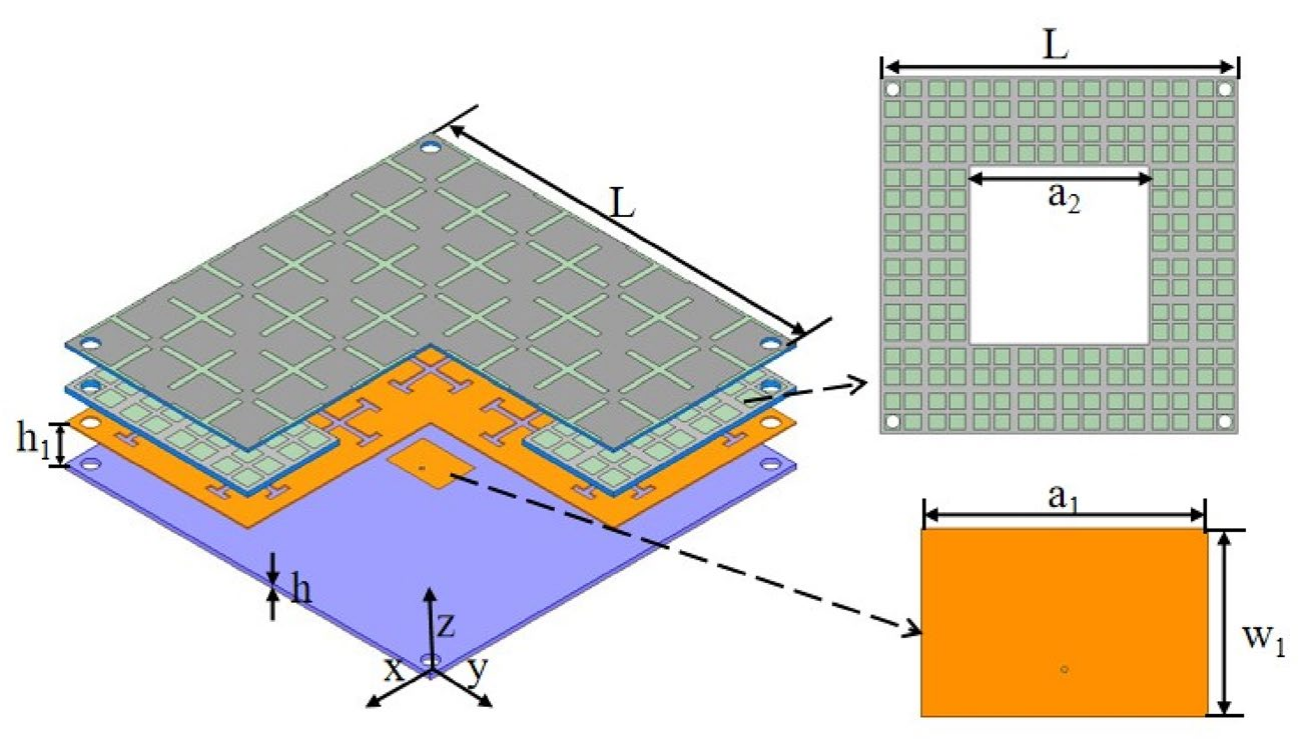
Structure of the proposed antenna. (Geometrical parameters: *L*=128 mm, $h_{1}$ = 15.4 mm, *h* = 1.6 mm, $a_{2}$ = 64 mm, $a_{1}$= 18.26 mm, $w_{1}$ = 13 mm).

The proposed antenna’s structure can be seen in Fig. 6. To guarantee the standard radiation of the antenna, a slot is cut with a width of $a_2*a_2$ in the second graphene lossy layer. A distance of $h_{1}$ is maintained between the patch antenna and the DGFSA superstrate. The dielectric board of the patch antenna is FR4, with dielectric constant of 4.4 and a thickness of *h*. The feed point of the proposed antenna is along the X-axis, 3mm separates the feed point from the radiation patch’s central location.

**Figure 7:**
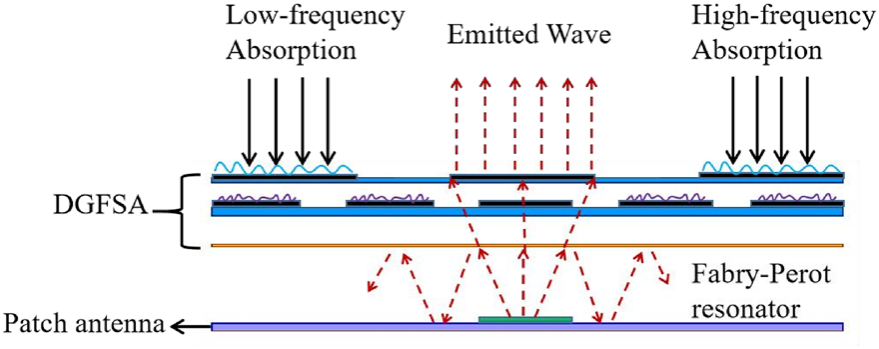
Functioning operation of the proposed antenna.

In Fig. 7, we can see the workings of the proposed antenna. DGFSA will absorb the incident electromagnetic waves at their out-of-band frequency. When the antenna work at the radiation frequency band, a quasi-Fabry-Perot resonator is created between the antenna’s ground and the DGFSA, enhancing the antenna’s gain.

### Radiation performance

**Figure 8:**
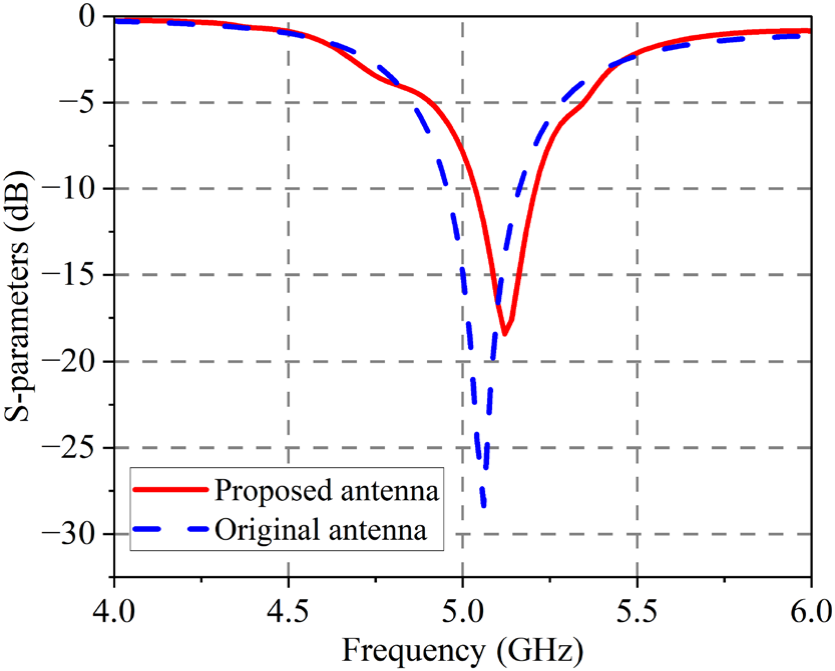
Simulated S-parameters of original and proposed antenna.

**Figure 9:**
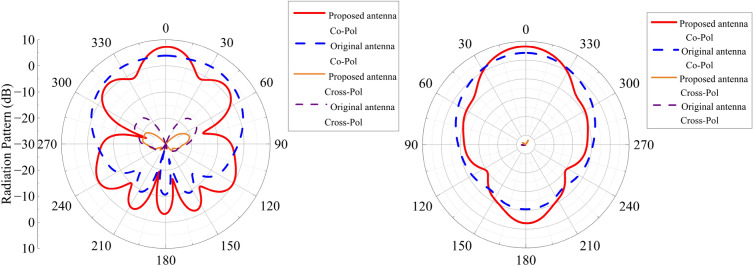
Simulated radiation patterns of proposed antenna and original antenna. (**a**) xoz plane (**b**) yoz plane.

A comparison of the antenna’s S-parameters with and without the DGFSA superstrate is presented in Fig. 8. The figure reveals that the proposed antenna operates at a frequency of 5.12 GHz, making it similar to the original antenna in this respect. The original antenna achieves an operating bandwidth of 4.95–5.16 GHz for a reflection coefficient $< -10$ dB, while the proposed antenna achieves it from 5.04 to 5.21 GHz with a little deviation from the original antenna. The antenna’s radiation pattern is shown in Fig. 9, the proposed antenna has a gain of 6.6 dB at 5.1 GHz, which is 2.4 dB higher than the original antenna due to the quasi-F-P effect.

### RCS performance

**Figure 10:**
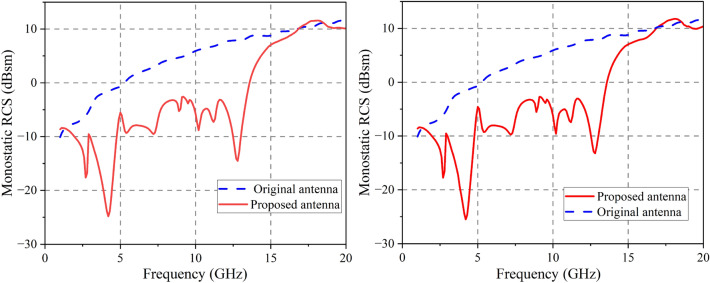
Simulated monostatic RCS of proposed and original antenna. (**a**) x-polarization (**b**) y-polarization.

Figure 10 shows the monostatic RCS of the original and proposed antennas when normal incident waves are present. The RCS of x- and y-polarized electromagnetic waves decreases dramatically from 1.4 to 16.8 GHz. The antenna has a reduction in RCS of 6 dB between 2.6 and 2.8 GHz when operated in x-polarization. (7.4%), 3.1–4.9 GHz (45%) and 5.1–14.0 GHz (93.19%). Under y-polarization, it achieves a 6 dB RCS reduction from 2.55–2.87 GHz (11.87%), 3.08–4.88 GHz (45.2%) and 5.18–13.89 GHz (91.35%). When dealing with electromagnetic waves with x and y polarization, the largest reduction of monostatic RCS is 23.28 dB around 4.2 GHz and 23.95 dB at 4.2 GHz, respectively.

**Figure 11:**
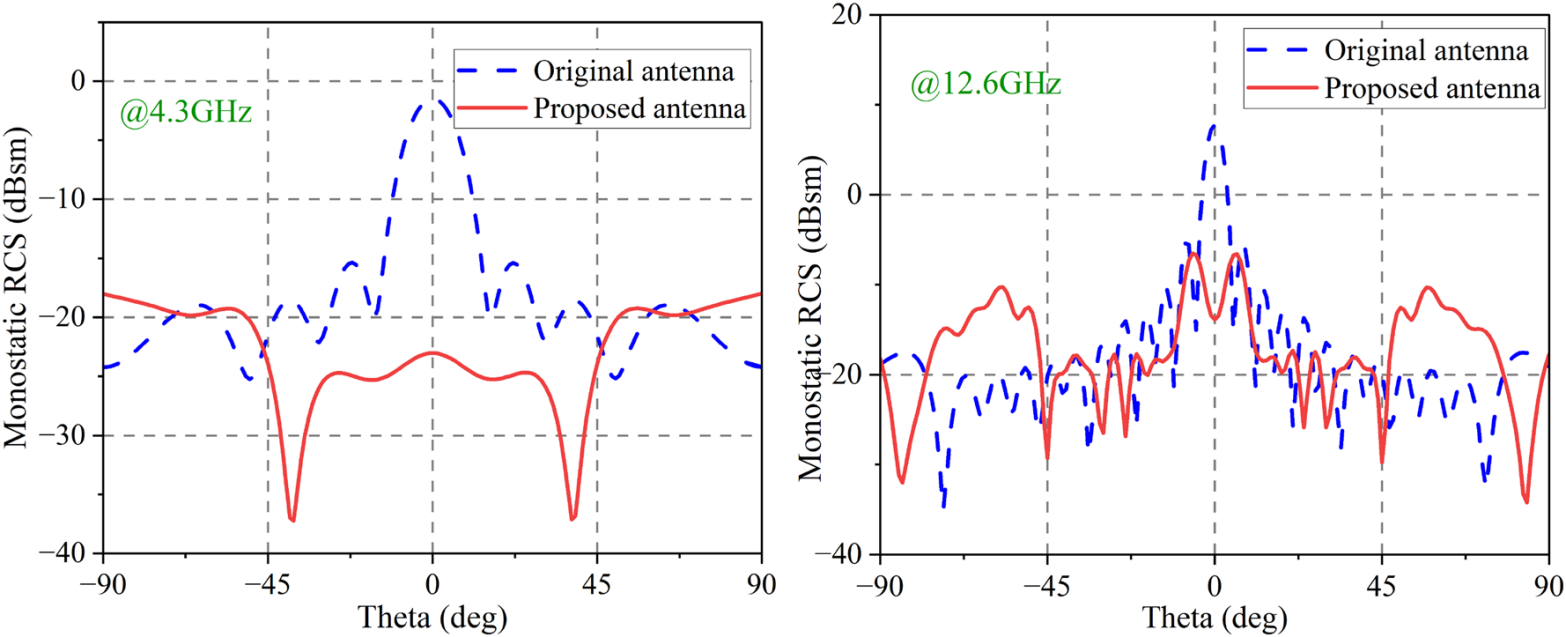
Simulated monostatic RCS for different EM incidence angles. (**a**) 4.3 GHz (**b**) 12.6 GHz.

Figure 11shows the simulated monostatic RCSs for different incidence angles at 4.3 GHz and 12.6 GHz, the antenna presented in this study achieves RCS reduction ($>0$ dB) over an angular range of about $\pm 45^{\circ }$ at 4.3 GHz. At 12.6 GHz, it can reduce RCS over an angular range of about $\pm 30^{\circ }$.

**Figure 12:**
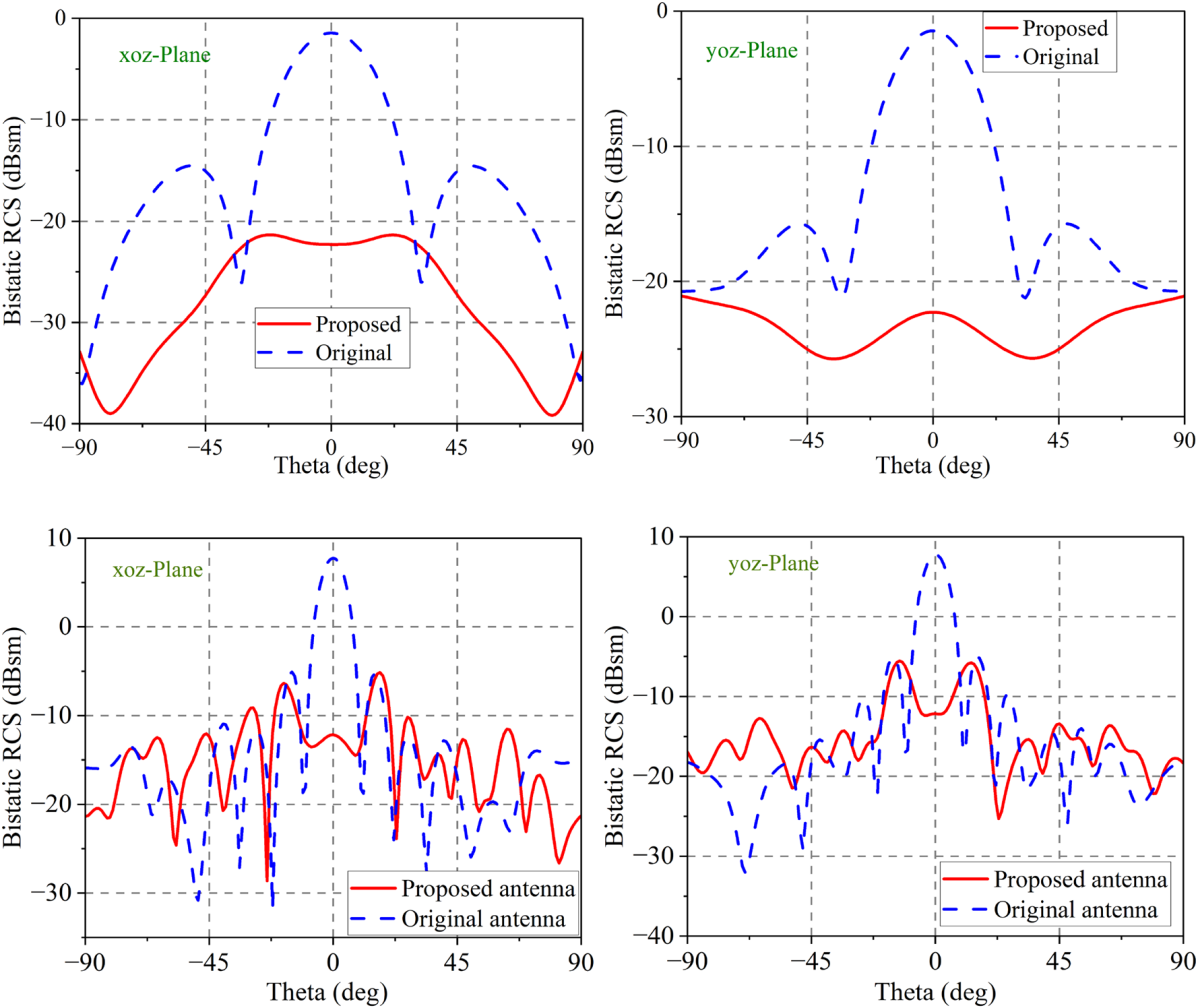
Bistatic RCS performance at different planes and frequencies (**a**,**b**) 4.3GHz (**c**,**d**) 12.6 GHz.

Compared in Fig. 12 are the bistatic RCSs performances of the original and proposed antenna designs. The antenna we proposed obtains significant RCS reduction over a wide angle compared to the original antenna. At 4.3 GHz, it is evident that the maximum RCS reduction in the xoz-plane is 22 dB, while it is 21 dB in the yoz-plane. In addition, it achieves plus and minus $80^{\circ }$ bistatic RCS reduction in the yoz-plane.

## Experimental verification

To ensure the proposed antenna’s radiation and scattering properties are accurate, a prototype is fabricated. The main difficulty of the antenna we proposed is the manufacturing of a graphene lossy layer. The proposed DGFSA is composed of two graphene lossy layers.

The top graphene is manufactured by the CVD process. First, the graphene monolayer is grown on the copper foil with a square resistance of 1200 $\Omega$ /sq, and then transfer the graphene to PET through the lamination and etching process. The designed square resistance of the second layer of graphene is 50 $\Omega$ /sq. In order to achieve the low square resistance of graphene, we grow eight graphene monolayers using the CVD process, by accumulating and transferring layer by layer onto PET, we finally get the square resistance we need.

**Figure 13:**
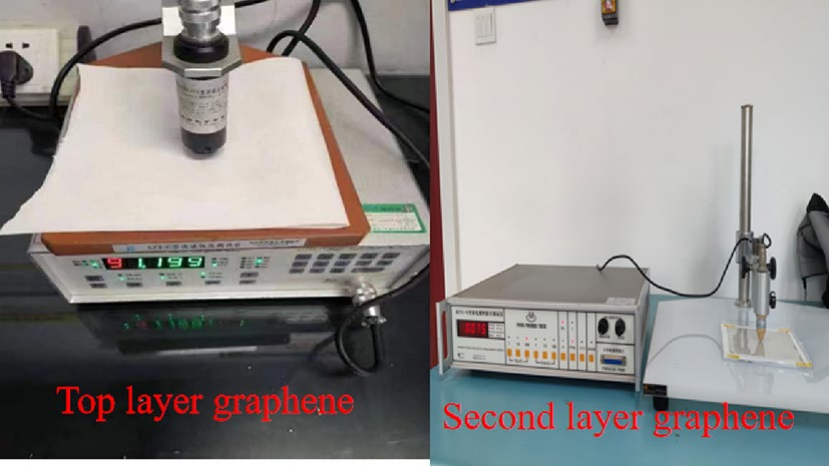
Measured square resistance of graphene.

**Figure 14:**
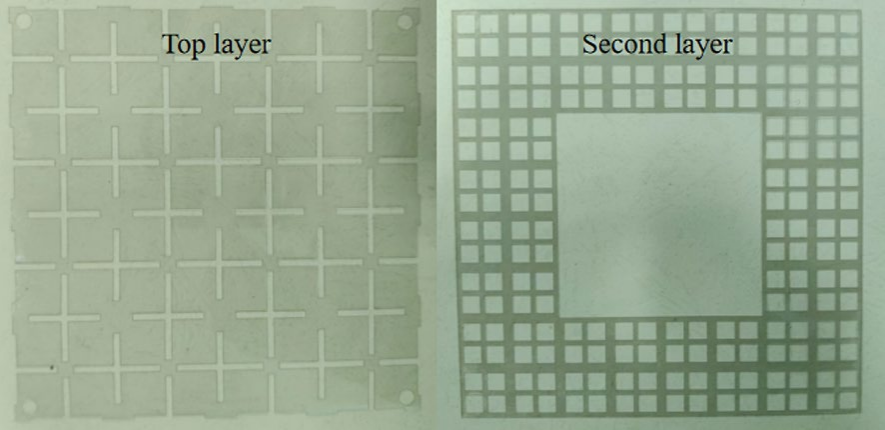
The top and, second layer of graphene.

**Figure 15:**
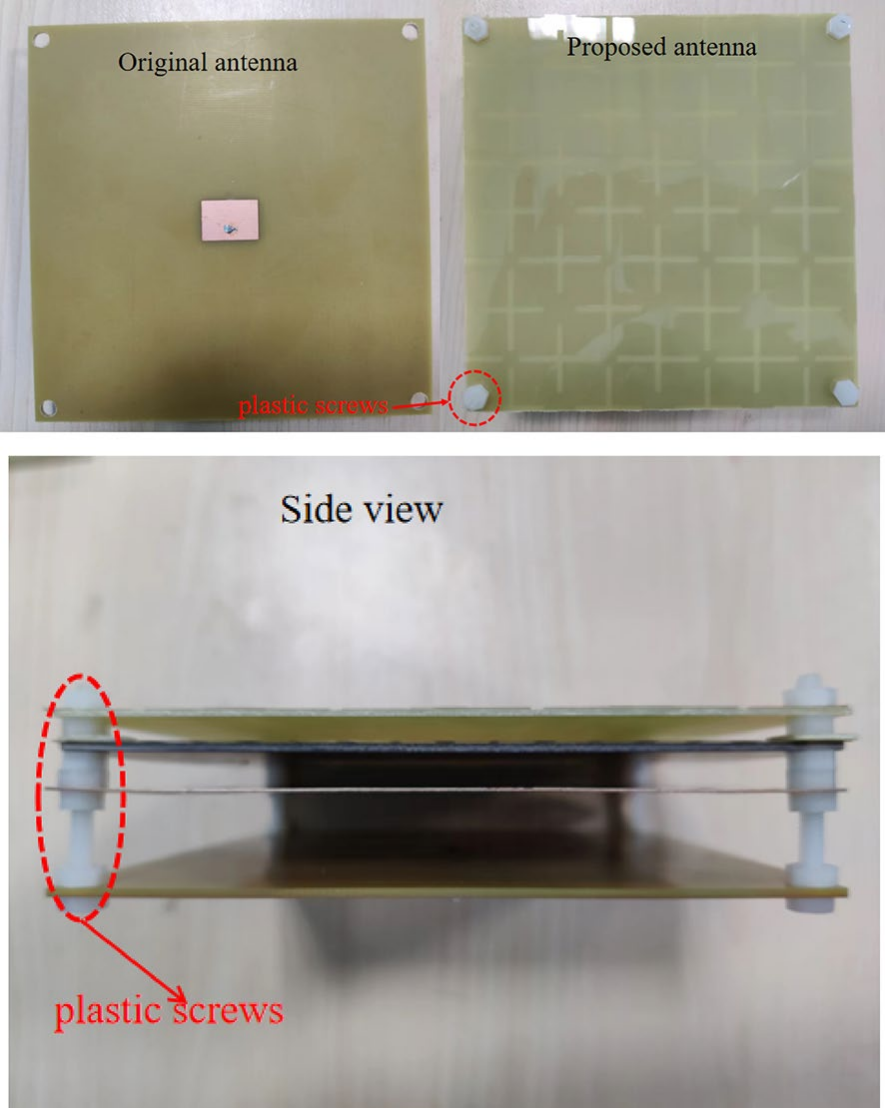
The structure of the proposed antenna. (**a**) Top view (**b**) Side view.

As shown in Fig. 13, we used a four-probe square resistance tester to evaluate the square resistance of the processed graphene. We can see that the square resistance of the top layer of graphene meets design requirements, because of the difficulty of accumulating and transferring, the square resistance of the second layer of graphene is 75 $\Omega$/sq. Finally, we use laser etching to get the graphene pattern we need The manufactured graphenes are pasted on the dielectric plate. The optimized parameters of the fabricated antenna are consistent with those proposed in sections “Design of Proposed DGFSA” and “Design of proposed low-RCS and gain enhancement antenna”. Figure 14 shows the top and, second layer graphene and Fig. 15 shows how the proposed antenna is constructed.

### Radiation performance

**Figure 16:**
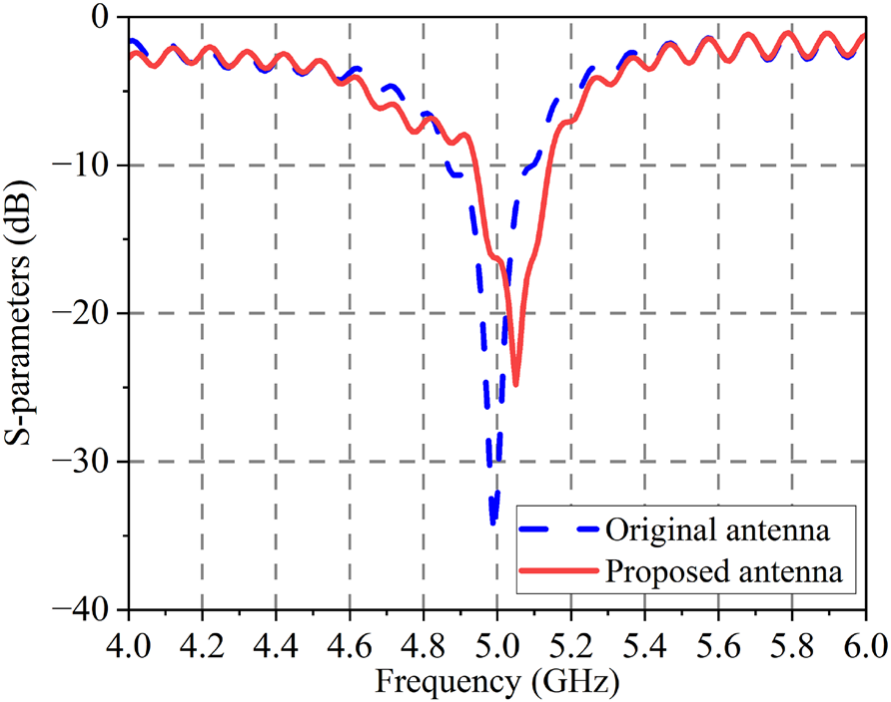
Measured |$S_{11}$| of proposed antenna.

Figure 16 shows the antenna’s measured |$S_{11}$| value. The working bandwidth of the antenna without or with DGFSA ranges from 4.91–5.11 GHz and 4.94–5.14 GHz. The discrepancy between the simulation and experiment is due to the processing of graphene and the assembly of the proposed antenna. As we all know, it is difficult to control the square resistance of graphene.

**Figure 17:**
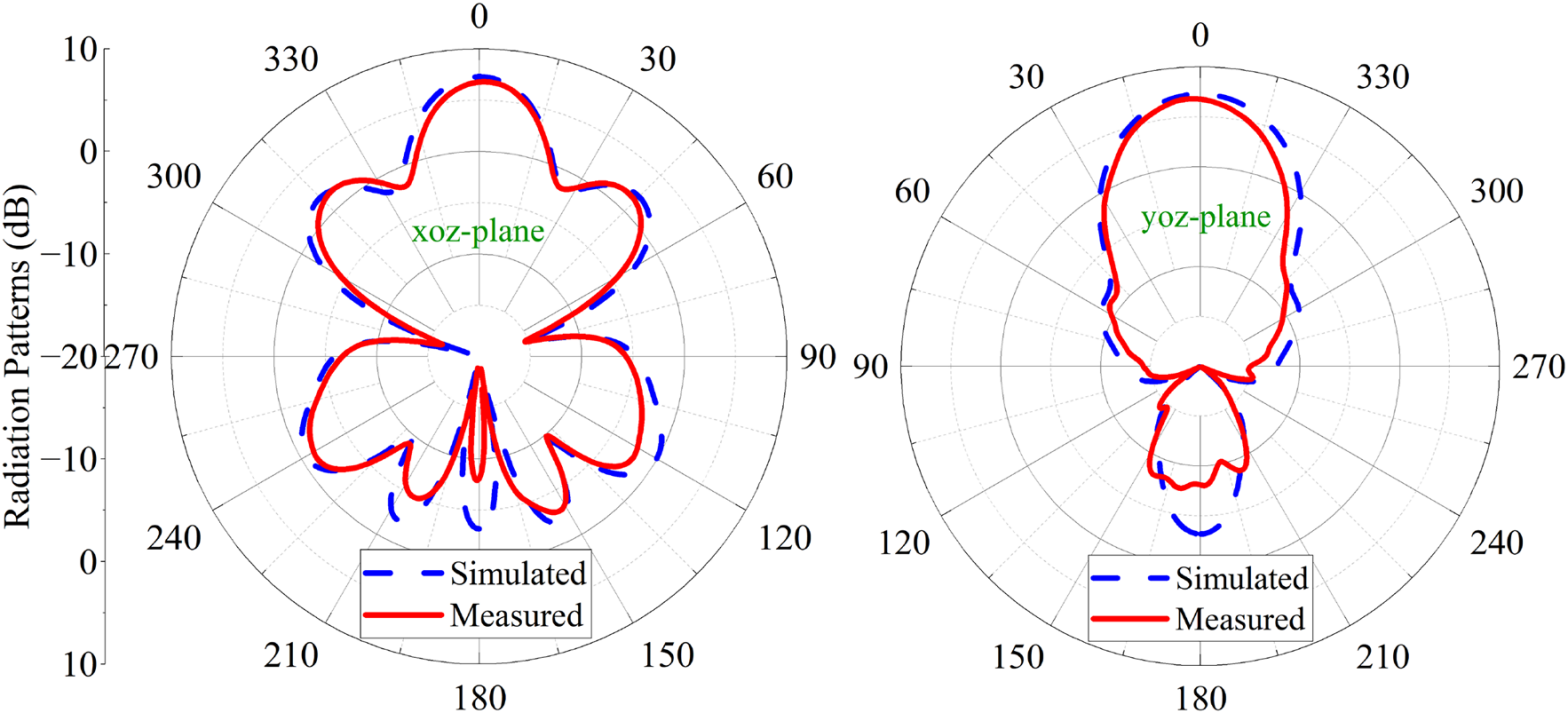
Simulated and measured radiation patterns of the proposed antenna. (**a**) xoz plane (**b**) yoz plane.

**Figure 18:**
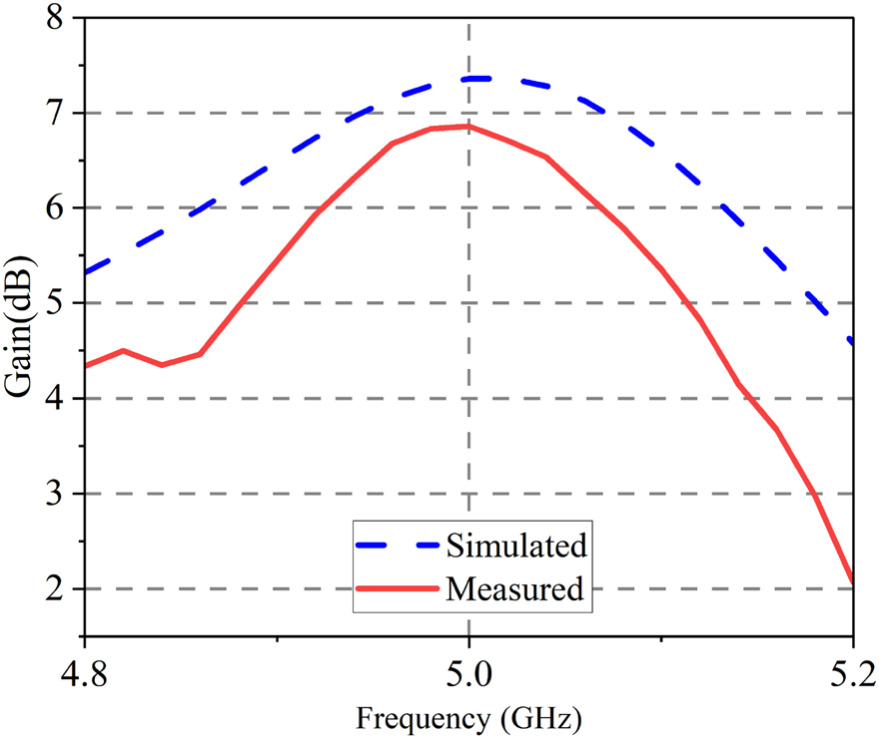
Gain of proposed antenna.

The radiation patterns at 5 GHz are presented in Fig. 17. The patterns of radiation show that the observed and simulated results correlate very well. The proposed antenna’s gain varies with frequency, as seen in Fig. 18. The max measured gain is obtained at 5GHz, which is 6.86 dB. The measured gain at 5 GHz drops 0.49 dB due to the processing of graphene and assembly of the proposed antenna.

### RCS performance

**Figure 19:**
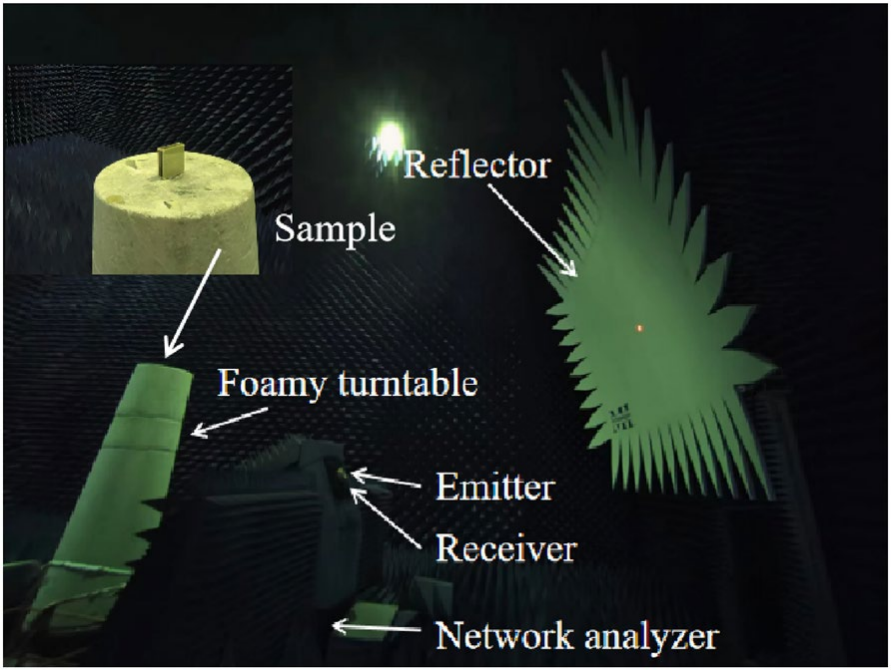
Test system for monostatic RCS in an anechoic chamber.

**Figure 20:**
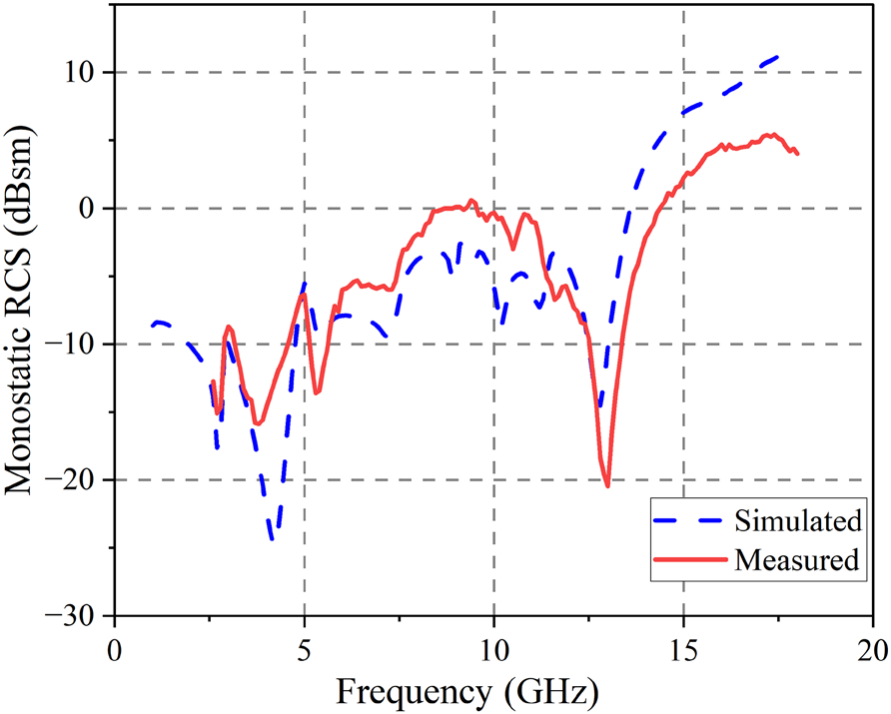
Monostatic RCS for x-polarization.

**Figure 21:**
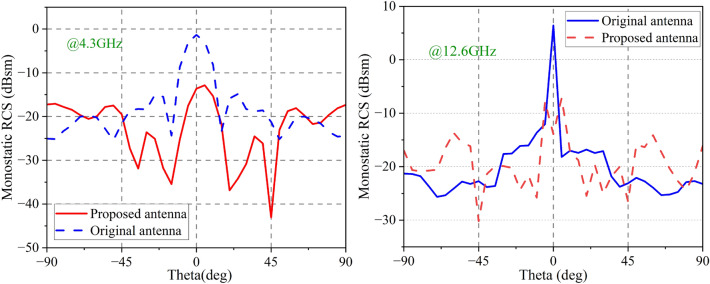
Monostatic RCS for different EM incidence angle.

An experimental setup for measuring the proposed antenna in a microwave anechoic chamber is depicted in Fig. 19. To cover the RCS reduction band, As emitters and receivers, five pairs of horn antennas operating in the bands 2.6–3.95, 3.95–5.85, 5.85–8.2, 8.2–12.4, and 12.4–18 GHz are employed. Monostatic RCS of the proposed antenna for normally x-polarized incident waves is shown in Fig. 20 along with a comparison to simulation findings. The simulated and experimental RCS values for the proposed antenna are consistent with one another. Figure 21 shows the measured monostatic RCSs for different incidence angles at 4.3 GHz and 12.6 GHz, the results agree with the simulated value. There are a number of potential causes for discrepancies between experimental and simulated results, such as (1) processing of graphene, (2) assembly of the proposed antenna, and (3) background noise of the measuring device in a microwave anechoic chamber.

**Table 1:** Comparison of proposed antenna and previous related work using metasurface absorber.

Reference/Year	Work frequency (GHz)	Antenna RCS reduction (dB)	RCS reduction bandwidth (GHz) and (%)	In band RCS reduction (dB)	Gain Enhancement (dB)	Lossy layer
2020	11.5–16.5	No	–	–	–	CVD graphene
2022	2.73–7.54	No	–	–	–	Graphene ink
2022	2.54–8.58	No	2–12 (Dual⌃2) (142.9%)	–	–	Graphene ink
2019	10.0	Yes	7–13 (Dual) (60%)	No	–	Lumped resistor
2022	4.8	Yes	4.4-18 (x-pol) (117.9%)	7.8	− 0.42	Lumped resistor
This work	5.12	Yes	1.4-16.8 (Dual) (169.2%)	5.62	2.4	CVD graphene

To show how valuable the proposed antenna is, we provide a comparison to related published research in Table 1. The majority of previous work about graphene-based absorbers is not used to reduce RCS^25,26^. In^28^, the graphene-based absorber is proposed to reduce the RCS of PEC, however, the paper only gives simulated data. Compared with previously reported low-RCS antennae using metasurface absorbers^30,31^, the antenna we proposed can achieve a more wide 6 dB RCS reduction bandwidth with gain enhancement.

## Conclusion

In this paper, using DGFSA, a wideband antenna that has low RCS and enhanced gain is proposed. Due to the wide-band EM wave absorption band of MGFSA and a quasi-Fabry-Perot effect, our proposed antenna shows a significant reduction in RCS between 1.32 and 17.13 GHz. (172.4$\%$), while its gain is improved by 2.3 dB simultaneously. Finally, the antenna is manufactured and evaluated. Results from simulations are highly correlated with those from experiments, which means the antenna we proposed has a good application on the stealth platform.

## Data Availability

The datasets generated during and/or analysed during the current study are available from the corresponding author on reasonable request.
